# Racial and Ethnic Differences in Subjective Cognitive Decline — United States, 2015–2020

**DOI:** 10.15585/mmwr.mm7210a1

**Published:** 2023-03-10

**Authors:** Karen G. Wooten, Lisa C. McGuire, Benjamin S. Olivari, Eva M. J. Jackson, Janet B. Croft

**Affiliations:** ^1^DB Consulting Group, Bethesda, Maryland; ^2^Division of Population Health, National Center for Chronic Disease Prevention and Health Promotion, CDC; ^3^Alzheimer’s Association, Chicago, Illinois; ^4^Oak Ridge Institute for Science and Education, Oak Ridge, Tennessee.

Subjective cognitive decline (SCD), the self-reported experience of worsening or more frequent memory loss or confusion, might be a symptom of early-stage dementia or future serious cognitive decline such as Alzheimer disease* or a related dementia (ADRD) (*1*). Established modifiable risk factors for ADRD include high blood pressure, inadequate physical activity, obesity, diabetes, depression, current cigarette smoking, and hearing loss (*2*). An estimated 6.5 million persons aged ≥65 years in the United States live with Alzheimer disease, the most common dementia (*1*). This number is projected to double by 2060, with the largest increase among non-Hispanic Black or African American (Black), and Hispanic or Latino (Hispanic) adults (*1*,*3*). Using data from the Behavioral Risk Factor Surveillance System (BRFSS), CDC assessed racial and ethnic, select demographic, and geographical differences in SCD prevalence, and prevalence of health care professional conversations among those reporting SCD. The age-adjusted prevalence of SCD during 2015–2020 was 9.6% among adults aged ≥45 years (5.0% of Asian or Pacific Islander [A/PI] adults, 9.3% of non-Hispanic White [White] adults, 10.1% of Black adults, 11.4% of Hispanic adults, and 16.7% of non-Hispanic American Indian or Alaska Native [AI/AN] adults). College education was associated with a lower prevalence of SCD among all racial and ethnic groups. Only 47.3% of adults with SCD reported that they had discussed confusion or memory loss with a health care professional. Discussing changes in cognition with a physician can allow for the identification of potentially treatable conditions, early detection of dementia, promotion of dementia risk reduction behaviors, and establishing a treatment or care plan to help adults remain healthy and independent for as long as possible.

BRFSS is a random-digit–dialed annual landline and cellular telephone cross-sectional survey of noninstitutionalized U.S. adults aged ≥18 years.^†^ The BRFSS six-question cognitive decline optional module (*4*) was administered to adults aged ≥45 years by all 50 states, Puerto Rico, and the District of Columbia at least once from 2015 to 2020; for states that implemented the module in multiple years, the most recent year of data was used.^§^ To maximize sample sizes for each racial and ethnic group,^¶^ data were aggregated for survey years 2015–2020. Overall telephone response rates** across survey years ranged from 45.9% (2017) to 49.4% (2019).

Respondents were classified as experiencing SCD if they responded “yes” when asked if they had experienced worsening or more frequent confusion or memory loss in the past 12 months; those who responded “yes” were asked if they had discussed SCD symptoms with a health care professional. Analyses were conducted using SAS-callable SUDAAN (version 11.0.3; RTI International) to account for complex sample survey design and weighting. Unadjusted and age-adjusted prevalence of SCD by race and ethnicity were estimated among 215,406 study participants aged ≥45 years overall, and for groups defined by jurisdiction, sex, age, education, marital status, health insurance status, and access to a personal doctor. Estimates that did not meet reliability standards (relative SE <30%) were suppressed. T-tests with p-values <0.05 were used to denote significant differences between racial and ethnic groups and differences between groups by selected characteristics. This study was reviewed by CDC and was conducted consistent with applicable federal law and CDC policy.^††^

Nearly 10% of adults aged ≥45 years (9.6%) reported experiencing SCD in the past 12 months (Table 1). SCD prevalence increased among successive age groups and was lower among adults with health insurance (9.5%) than among those without health insurance (11.6%) and decreased with increasing formal educational attainment. The highest overall percentages of SCD occurred among adults who did not have a high school diploma (16.4%), those who had been married, but were not currently married (13.6%), and those aged ≥75 years (13.3%). Age-adjusted SCD was higher among AI/AN (16.7%) and Hispanic adults (11.4%) than among White adults (9.3%) and was lower among A/PI adults (5.0%); prevalence among Black adults (10.1%) was similar to that among White adults (9.3%). This pattern of racial and ethnic differences was observed across most demographic subcategories examined. Unadjusted overall prevalence of SCD was highest in Alabama (14.3%), Oklahoma (14.1%), Florida (13.6%), Louisiana (13.6%), West Virginia (13.6%), Tennessee (12.9%), and New Mexico (12.8%), and lowest in Illinois 6.1% (Table 2). Because of small sample sizes, estimates for A/PI and AI/AN adults were unstable in most states.

**TABLE 1:** Prevalence* of subjective cognitive decline^†^ among adults aged ≥45 years, by race and ethnicity and selected characteristics — Behavioral Risk Factor Surveillance System, United States,^§^ 2015–2020.

Characteristic	Race and ethnicity^¶^					
	Adults reporting subjective cognitive decline, % (95% CI)					
	Total (N = 215,406)**	American Indian or Alaska Native (n = 3,500)	Asian or Pacific Islander (n = 3,427)	Black or African American (n = 14,700)	White (n = 172,437)	Hispanic or Latino (n = 11,961)
**Overall**						
Unadjusted	**9.7 (9.3–10.0)**	16.4 (13.0–20.4)^††^	4.1 (3.0–5.7)^††^	10.0 (9.1–10.9)	9.5 (9.1–9.8)	11.2 (9.8–12.8)
Age-adjusted	**9.6 (9.3–10.0)**	16.7 (13.0–21.2)^††^	5.0 (3.7–6.6)^††^	10.1 (9.2–11.1)	9.3 (8.9–9.7)	11.4 (10.0–13.1)^††^
**Sex**						
Men	**9.3 (8.9–9.8)**	16.2 (11.8–21.8)^††^	5.9 (4.0–8.6)^††^	9.4 (8.1–10.9)	9.3 (8.8–9.9)	9.1 (7.5–11.0)
Women^§§^	**9.9 (9.4–10.4)**	17.5 (12.1–24.6)^††^	4.3 (2.8–6.6)^††^	10.7 (9.5–12.0)	9.3 (8.8–9.8)	13.4 (11.2–16.0)^††,¶¶^
**Age group, yrs**						
45–64^§§^	**8.8 (8.5–9.2)**	18.6 (14.0–24.1)^††^	3.7 (2.5–5.5)^††^	9.5 (8.4–10.7)	8.4 (8.0–8.8)	10.5 (9.0–12.3)^††^
65–74	**9.5 (8.8–10.2)**	11.9 (8.3–16.9)	—***	11.4 (9.5–13.7)	9.0 (8.4–9.6)	12.4 (8.5–17.6)
≥75	**13.3 (12.3–14.4)^¶¶^**	—	8.9 (5.2–14.7)	11.5 (9.5–13.8)	13.4 (12.2–14.6)^¶¶^	14.1 (10.0–19.5)
**Education level**						
Not high school graduate	**16.4 (15.0–17.9)^¶¶^**	26.1 (16.9–37.9)^¶¶^	—	19.1 (15.4–23.4)^¶¶^	19.2 (17.3–21.1)^¶¶^	13.0 (10.4–16.0)^††,¶¶^
High school graduate	**10.6 (10.0–11.3)^¶¶^**	13.9 (9.0–20.8)^††,¶¶^	—	10.8 (9.3–12.5)^¶¶^	10.3 (9.6–11.0)^¶¶^	12.6 (9.7–16.3)^¶¶^
Some college	**9.4 (8.8–10.1)^¶¶^**	17.4 (13.1–22.8)^††,¶¶^	4.1 (2.5–6.7)^††^	7.9 (6.6–9.3)	9.6 (8.8–10.4)^¶¶^	10.6 (7.6–14.6)
College graduate^§§^	**6.3 (5.9–6.7)**	6.6 (4.2–10.3)	3.9 (2.5–6.0)	6.8 (5.3–8.7)	6.2 (5.8–6.6)	7.2 (5.5–9.4)
**Marital status**						
Currently married^§§^	**7.7 (7.3–8.1)**	15.8 (11.6–21.1)^††^	4.4 (3.0–6.4)^††^	8.0 (6.7–9.5)	7.5 (7.1–7.9)	9.2 (7.3–11.6)
Once married, but not currently married	**13.6 (12.9–14.3)^¶¶^**	19.5 (12.6–28.9)	8.0 (4.7–13.4)	12.2 (10.6–14.0)^¶¶^	13.6 (12.8–14.5)^¶¶^	14.5 (12.2–17.2)^¶¶^
Never married	**11.6 (10.4–13.0)^¶¶^**	13.5 (7.7–22.6)	—	10.4 (8.2–13.0)	11.3 (9.9–12.9)^¶¶^	16.3 (11.0–23.6)
**Health insurance status**						
Has insurance^§§^	**9.5 (9.1–9.8)**	16.8 (12.8–21.7)^††^	4.4 (3.2–6.0)^††^	9.8 (8.9–10.8)	9.1 (8.7–9.5)	11.8 (10.2–13.7)^††^
Does not have insurance	**11.6 (10.1–13.3)^¶¶^**	15.2 (9.1–24.3)	23.3 (15.2–34.0)^††,¶¶^	11.7 (8.5–15.9)	11.7 (10.2–13.4)^¶¶^	11.5 (7.2–18.0)
**Has personal doctor**						
Yes^§§^	**9.6 (9.3–10.0)**	17.8 (13.3–23.3)	5.1 (3.8–6.9)	9.7 (8.8–10.7)	9.3 (8.9–9.7)	12.0 (10.3–13.8)
No	**9.8 (8.8–11.0)**	15.6 (10.7–22.2)	—	13.4 (10.1–17.7)	9.3 (8.3–10.5)	9.7 (6.5–14.2)
**Visited doctor in preceding year**						
Yes^§§^	**10.0 (9.6–10.3)**	18.8 (14.4–24.3)^††^	5.7 (4.2–7.7)^††^	10.1 (9.1–11.1)	9.6 (9.2–10.0)	12.0 (10.3–13.9)^††^
No	**8.2 (7.1–9.5)^¶¶^**	9.2 (5.6–14.5)	—	10.3 (7.5–13.9)	8.2 (6.9–9.6)	10.3 (6.1–16.9)

* Except for age-specific and overall unadjusted estimates, estimates were age-standardized to the 2000 projected U.S. Census Bureau population aged ≥45 years using three age groups: 45–64, 65–74, and ≥75 years. https://www.cdc.gov/nchs/data/statnt/statnt20.pdf

^†^ Defined as the self-reported experience of worsening confusion or memory loss in the preceding year.

^§^ Estimates are aggregated to include the most recent survey year for each jurisdiction. These estimates include data collected from jurisdictions in 2020 (Alaska, Arizona, Arkansas, California, Delaware, District of Columbia, Hawaii, Idaho, Illinois, Kentucky, Maine, Michigan, Nevada, New Hampshire, New York, North Carolina, Ohio, Oregon, Utah, Vermont, Washington, Wyoming, and Puerto Rico); 2019 (Alabama, Connecticut, Florida, Georgia, Indiana, Iowa, Kansas, Louisiana, Maryland, Minnesota, Mississippi, Missouri, Nebraska, New Mexico, North Dakota, Oklahoma, Pennsylvania, Rhode Island, South Carolina, South Dakota, Tennessee, Texas, Virginia, West Virginia, and Wisconsin); 2018 (New Jersey); 2016 (Massachusetts and Montana); and 2015 (Colorado).

^¶^ Persons of Hispanic or Latino (Hispanic) origin might be of any race but are categorized as Hispanic; all racial groups are non-Hispanic.

** Total study population includes respondents in the five racial and ethnic groups (206,025) and an additional 9,381 adults reporting multiple races and other race categories.

^††^ The estimate for this racial and ethnic group differs statistically from that of White adults (p<0.05).

^§§^ Reference group for comparisons for groups defined by a characteristic within the overall population and within each racial and ethnic group.

^¶¶^ The estimate for this group differs statistically from the reference group defined by this characteristic (p<0.05).

*** Dashes indicate estimate is not reported because relative SE >30% or sample size <50.

**TABLE 2:** Unadjusted prevalence of subjective cognitive decline* among adults aged ≥45 years, by race and ethnicity and jurisdiction^†^ — Behavioral Risk Factor Surveillance System, United States, 2015–2020

Jurisdiction	Race and ethnicity^§^					
	Adults reporting subjective cognitive decline, % (95% CI)					
	Total 2015–2020	American Indian or Alaska Native	Asian or Pacific Islander	Black or African American	White	Hispanic or Latino
Alabama	**14.3 (12.9–15.7)**	29.9 (16.8–47.3)^¶^	—**	13.8 (11.4–16.8)	14.2 (12.6–15.9)	—
Alaska	**8.6 (6.8–10.7)**	6.2 (3.6–10.6)	—	—	7.7 (6.0–9.8)	—
Arizona	**8.8 (7.9–9.9)**	10.6 (6.7–16.4)	—	—	8.6 (7.5–9.8)	9.3 (7.1–12.2)
Arkansas	**11.5 (10.1–13.0)**	—	—	10.9 (7.5–15.4)	11.8 (10.3–13.5)	—
California	**7.3 (5.5–9.6)**	—	—	—	7.8 (5.3–11.2)	10.8 (7.0–16.4)
Colorado	**10.7 (9.5–12.1)**	—	—	—	9.7 (8.4–11.1)	16.6 (12.0–22.4)^¶^
Connecticut	**7.3 (6.5–8.2)**	—	—	9.1 (6.0–13.6)	6.9 (6.1–7.8)	8.9 (5.6–14.0)
Delaware	**8.2 (6.8–9.9)**	—	—	8.2 (5.3–12.4)	8.0 (6.4–10.0)	—
District of Columbia	**8.8 (7.1–10.8)**	—	—	10.2 (7.9–13.0)^¶^	5.6 (4.1–7.5)	—
Florida	**13.6 (12.2–15.1)**	—	—	9.7 (6.6–14.0)	14.1 (12.7–15.6)	14.3 (9.8–20.4)
Georgia	**10.9 (9.6–12.4)**	—	—	8.9 (6.5–12.0)	11.6 (10.0–13.3)	—
Hawaii	**6.7 (5.8–7.7)**	—	6.1 (4.8–7.8)	—	7.3 (5.8–9.2)	8.8 (5.7–13.2)
Idaho	**9.2 (7.9–10.8)**	—	—	—	8.8 (7.5–10.4)	—
Illinois	**6.1 (4.8–7.7)**	—	—	—	5.8 (4.4–7.6)	—
Indiana	**11.2 (10.2–12.2)**	—	—	11.8 (8.3–16.5)	10.8 (9.8–11.8)	13.3 (7.3–23.1)
Iowa	**10.0 (9.1–10.9)**	—	—	—	9.8 (9.0–10.7)	11.4 (6.5–19.1)
Kansas	**11.5 (10.2–12.8)**	—	—	—	11.2 (9.9–12.5)	—
Kentucky	**11.2 (9.6–13.0)**	—	—	—	11.6 (10.0–13.5)	—
Louisiana	**13.6 (12.1–15.3)**	—	—	16.4 (13.0–20.5)	12.3 (10.6–14.2)	—
Maine	**7.9 (7.0–8.8)**	—	—	—	7.9 (7.0–8.8)	—
Maryland	**10.1 (8.9–11.4)**	—	—	9.2 (7.0–11.9)	10.9 (9.5–12.6)	—
Massachusetts	**9.3 (8.2–10.5)**	—	—	—	8.2 (7.0–9.4)	16.5 (11.4–23.2)^¶^
Michigan	**9.5 (7.6–11.7)**	—	—	11.0 (6.1–18.9)	8.2 (6.5–10.3)	—
Minnesota	**8.9 (8.1–9.7)**	—	—	9.5 (6.0–14.8)	8.7 (8.0–9.5)	5.8 (3.2–10.2)
Mississippi	**11.8 (10.4–13.4)**	—	—	13.2 (10.7–16.1)	10.1 (8.6–11.9)	—
Missouri	**11.2 (10.0–12.5)**	—	—	8.3 (5.5–12.4)	11.2 (10.0–12.6)	—
Montana	**9.8 (8.6–11.2)**	16.4 (10.7–24.3)^¶^	—	—	9.0 (7.8–10.3)	—
Nebraska	**9.6 (8.5–10.7)**	—	—	—	9.2 (8.2–10.3)	5.9 (3.4–10.1)
Nevada	**10.3 (8.0–13.2)**	—	—	—	9.8 (7.5–12.8)	—
New Hampshire	**6.9 (6.1–8.0)**	—	—	—	6.3 (5.5–7.3)	—
New Jersey	**8.8 (6.9–11.2)**	—	—	—	8.9 (6.6–11.9)	—
New Mexico	**12.8 (11.4–14.4)**	13.1 (8.7–19.3)	—	—	11.2 (9.6–13.2)	15.0 (12.4–18.1)
New York	**7.1 (5.8–8.7)**	—	—	5.4 (3.1–9.2)	5.8 (4.5–7.4)	13.8 (8.2–22.2)
North Carolina	**6.9 (5.9–8.2)**	24.9 (15.0–38.4)	—	5.7 (4.0–8.3)	7.1 (5.8–8.7)	—
North Dakota	**8.1 (7.0–9.3)**	—	—	—	7.5 (6.6–8.6)	—
Ohio	**9.1 (8.1–10.1)**	—	—	12.4 (8.3–18.1)	8.2 (7.3–9.3)	—
Oklahoma	**14.1 (12.2–16.2)**	13.9 (7.8–23.5)	—	—	14.2 (12.1–16.5)	—
Oregon	**9.5 (8.2–11.1)**	—	—	—	8.9 (7.8–10.2)	9.1 (5.0–16.0)
Pennsylvania	**9.6 (8.4–10.9)**	—	—	14.7 (10.3–20.6)	8.8 (7.7–10.1)	—
Puerto Rico	**6.2 (4.7–8.3)**	—	—	—	—	6.3 (4.7–8.4)
Rhode Island	**10.2 (9.0–11.6)**	—	—	—	9.5 (8.3–10.9)	21.4 (14.0–31.3)^¶^
South Carolina	**11.5 (10.3–12.8)**	—	—	12.3 (9.4–15.8)	11.0 (9.7–12.5)	—
South Dakota	**9.5 (7.8–11.6)**	—	—	—	8.5 (7.0–10.4)	—
Tennessee	**12.9 (11.6–14.5)**	—	—	16.5 (11.7–22.6)	12.1 (10.7–13.6)	—
Texas	**11.6 (10.3–13.2)**	—	—	15.9 (10.4–23.5)	10.9 (9.3–12.7)	12.4 (9.7–15.8)
Utah	**9.8 (8.6–11.2)**	—	—	—	10.6 (9.2–12.1)	—
Vermont	**7.5 (6.5–8.6)**	—	—	—	7.4 (6.4–8.6)	—
Virginia	**9.5 (8.6–10.5)**	—	—	11.0 (8.6–13.8)	9.3 (8.3–10.4)	—
Washington	**9.3 (8.5–10.3)**	—	7.3 (4.3–12.2)	11.4 (6.3–19.7)	9.2 (8.3–10.1)	9.4 (5.7–15.0)
West Virginia	**13.6 (12.3–15.0)**	—	—	—	13.5 (12.2–15.0)	—
Wisconsin	**9.6 (8.4–11.0)**	—	—	—	9.8 (8.5–11.2)	—
Wyoming	**8.0 (6.8–9.4)**	—	—	—	7.8 (6.6–9.2)	—

* Subjective cognitive decline was defined as the self-reported experience of worsening confusion or memory loss in the preceding year.

^†^ Estimates are aggregated to include the most recent survey year for each jurisdiction. These estimates include data collected from jurisdictions in 2020 (Alaska, Arizona, Arkansas, California, Delaware, District of Columbia, Hawaii, Idaho, Illinois, Kentucky, Maine, Michigan, Nevada, New Hampshire, New York, North Carolina, Ohio, Oregon, Utah, Vermont, Washington, Wyoming, and Puerto Rico); 2019 (Alabama, Connecticut, Florida, Georgia, Indiana, Iowa, Kansas, Louisiana, Maryland, Minnesota, Mississippi, Missouri, Nebraska, New Mexico, North Dakota, Oklahoma, Pennsylvania, Rhode Island, South Carolina, South Dakota, Tennessee, Texas, Virginia, West Virginia, and Wisconsin); 2018 (New Jersey); 2016 (Massachusetts and Montana); and 2015 (Colorado).

^§^ Persons of Hispanic or Latino (Hispanic) origin might be of any race but are categorized as Hispanic; all racial groups are non-Hispanic.

^¶^ The estimate for the selected racial and ethnic group differs statistically from that of White adults (p<0.05) within each jurisdiction.

** Dashes indicate estimate is not reported because relative SE >30% or sample size <50.

Among 21,299 respondents with SCD, 47.3% reported talking with a health care professional about confusion or memory loss; women (50.7%) were more likely than men (43.3%) to do so (Table 3). Overall and within racial and ethnic groups, adults with SCD symptoms who were less likely to talk to a health care professional about their symptoms were aged ≥75 years, had less education, did not have health insurance, did not have a personal doctor, and had not visited a doctor in the past year.

**TABLE 3:** Percentage* of adults aged ≥45 years with subjective cognitive decline**^†^** who talked about confusion or memory loss with a health care professional, by race and ethnicity and other selected characteristics — Behavioral Risk Factor Surveillance System, United States,**^§^** 2015–2020.

Characteristic	Race and ethnicity^¶^					
	Adults reporting subjective cognitive decline who spoke with health care professional, % (95% CI)					
	Total (N = 21,299)**	American Indian or Alaska Native (n = 493)	Asian or Pacific Islander (n = 212)	Black or African American (n = 1,588)	White (n = 16,700)	Hispanic or Latino (n = 1,224)
**Overall**						
Unadjusted	**46.0 (44.2–47.8)**	51.7 (39.8–63.4)	35.3 (22.9–50.1)	49.4 (44.8–54.1)	45.9 (44.0–47.8)	41.8 (34.8–49.3)
Age-adjusted	**47.3 (45.5–49.1)**	50.5 (39.0–61.9)	34.5 (22.6–48.9)	49.5 (44.7–54.4)	48.5 (46.6–50.4)	40.5 (33.9–47.4)
**Sex**						
Men	**43.3 (40.7–46.0)^††^**	51.8 (36.8–66.5)	35.6 (21.0–53.5)	43.4 (35.6–51.5)	44.0 (41.0–47.0)^††^	40.9 (31.9–50.6)
Women^§§^	**50.7 (48.2–53.1)**	49.9 (38.1–61.8)	32.2 (18.6–49.7)^¶¶^	54.2 (48.4–59.8)	52.4 (49.8–54.9)	40.4 (32.1–49.4)^¶¶^
**Age group, yrs**						
45–64^§§^	**50.6 (48.3-52.8)**	55.9 (41.4–69.4)	33.3 (19.1–51.4)	54.8 (48.6–60.8)	51.8 (49.4–54.1)	41.6 (33.9–49.7)
65–74	**48.6 (44.8-52.5)**	44.6 (30.1–60.0)	—***	46.0 (36.2–56.2)	48.7 (45.1–52.2)	49.3 (30.1–68.7)
≥75	**34.0 (30.7-37.5)^††^**	—	—	32.7 (24.8-41.7)^††^	34.1 (30.3-38.1)^††^	32.2 (18.8-49.3)
**Education level**						
Not high school graduate	**41.4 (36.8–46.2)^††^**	49.6 (31.5–67.8)	—	42.8 (33.1–53.0)	44.0 (39.3–48.8)	34.4 (24.3–46.1)
High school graduate	**43.4 (40.4–46.4)^††^**	37.0 (25.7–49.9)^††^	—	46.1 (39.1–53.3)	43.9 (40.6–47.3)^††^	44.1 (34.4–54.3)
Some college	**54.1 (51.0–57.2)**	63.2 (49.8–74.8)	56.4 (39.1–72.2)^††^	54.2 (46.1–62.1)	53.5 (49.9–57.1)	53.9 (41.6–65.8)
College graduate^§§^	**50.2 (46.9–53.4)**	66.7 (51.6–79.0)	23.0 (13.7–36.0)^¶¶^	62.3 (51.5–72.0)	50.9 (47.4–54.3)	45.8 (32.9–59.2)
**Marital status**						
Currently married^§§^	**46.1 (43.4–48.7)**	53.5 (39.9–66.6)	32.6 (18.6–50.6)	50.3 (40.6–60.1)	47.8 (45.1–50.5)	36.5 (27.1–47.1)
Divorced or widowed	**48.8 (46.0–51.5)**	52.2 (41.0–63.2)	42.8 (26.7–60.6)	50.1 (43.5–56.7)	49.1 (45.9–52.4)	43.2 (34.4–52.4)
Never married	**51.4 (45.7–57.1)**	34.6 (19.7–53.3)	—	57.6 (47.5–67.2)	50.5 (44.1–56.9)	63.7 (47.9–77.1)^††^
**Health insurance status**						
Has insurance^§§^	**48.7 (46.8–50.7)**	51.8 (39.6–63.7)	36.5 (23.8–51.4)	53.1 (47.7–58.3)	49.2 (47.2–51.3)	42.5 (35.2–50.2)
Does not have insurance	**32.5 (27.8–37.7)^††^**	—	—	23.4 (14.5–35.6)^††^	39.7 (33.2–46.6)^††^	23.5 (15.5–34.0)^††^
**Has personal doctor**						
Yes^§§^	**49.9 (48.0–51.9)**	52.6 (40.2–64.7)	36.5 (23.8–51.5)	52.2 (46.8–57.6)	50.6 (48.5–52.7)	44.8 (37.7–52.2)
No	**30.4 (25.6–35.5)^††^**	40.2 (25.0–57.5)	—	38.4 (26.6–51.6)	31.2 (26.0–36.9)^††^	27.5 (19.3–37.6)^††^
**Doctor visit in preceding year**						
Yes^§§^	**50.7 (48.7–52.7)**	51.6 (39.3–63.7)	42.8 (29.9–56.8)	51.8 (46.5–57.1)	52.3 (50.1–54.4)	44.7 (37.3–52.3)
No	**27.2 (23.7–31.1)^††^**	35.0 (20.3–53.2)	—	34.0 (23.9–45.8)^††^	27.9 (24.3–31.9)^††^	16.7 (9.2–28.3)^††^

* Except for age-specific and overall unadjusted estimates, estimates were age-standardized to the 2000 projected U.S. Census Bureau population aged ≥45 years using three age groups: 45–64, 65–74, and ≥75 years. https://www.cdc.gov/nchs/data/statnt/statnt20.pdf

^† ^Defined as the self-reported experience of worsening confusion or memory loss in the preceding year.

^§^ Estimates are aggregated to include the most recent survey year for each jurisdiction. These estimates include data collected from jurisdictions in 2020 (Alaska, Arizona, Arkansas, California, Delaware, District of Columbia, Hawaii, Idaho, Illinois, Kentucky, Maine, Michigan, Nevada, New Hampshire, New York, North Carolina, Ohio, Oregon, Utah, Vermont, Washington, Wyoming, and Puerto Rico); 2019 (Alabama, Connecticut, Florida, Georgia, Indiana, Iowa, Kansas, Louisiana, Maryland, Minnesota, Mississippi, Missouri, Nebraska, New Mexico, North Dakota, Oklahoma, Pennsylvania, Rhode Island, South Carolina, South Dakota, Tennessee, Texas, Virginia, West Virginia, and Wisconsin); 2018 (New Jersey); 2016 (Massachusetts and Montana); and 2015 (Colorado).

^¶^ Persons of Hispanic or Latino (Hispanic) origin might be of any race but are categorized as Hispanic; all racial groups are non-Hispanic.

** Total study population includes those in the five racial and ethnic groups (N = 20,217) and 1,082 adults reporting multiple races and other race categories.

^††^ Estimate for this group differs statistically from the reference group for this characteristic (p<0.05).

^§§^ Reference group.

^¶¶^ Estimate for the selected racial and ethnic group differs statistically from that of White adults (p<0.05).

*** Dashes indicate estimate suppressed (relative SE >30% or sample size <50).

## Discussion

Prevalence of SCD varied across adults by demographic characteristics and race and ethnicity. Among racial and ethnic groups, SCD was lowest among A/PI adults and highest among AI/AN adults. Prevalence of SCD was higher among persons with less formal education than among college graduates across all racial and ethnic groups. This finding is consistent with other studies that suggest that persons with more years of formal education have a lower risk for dementia than do those with fewer years of formal education (*5*,*6*). Low prevalence of SCD among adults with higher education suggests that education might be protective against SCD. More research is needed to better understand the roles that education and related systemic factors play in sustaining cognitive health, particularly across diverse racial and ethnic populations. For example, modifiable risk factors for ADRD are less prevalent among adults with higher education, differ among racial and ethnic groups, and are associated with high prevalence of SCD (*2*).

The findings of this study can help health care providers identify groups of patients who would benefit from risk reduction behaviors and further cognitive assessment. Persons who talked with a health care professional about SCD were more likely to be women, had at least some college education, were aged <75 years, had a personal doctor, had a doctor visit within the past year, and had health insurance. Public health strategies are needed to support access to health care for persons who lack access to routine health care or to have a designated preventive health care professional. For example, programs such as Welcome to Medicare^§§^ and Medicare Annual Wellness Visit for adults^¶¶^ aged >65 provide coverage for preventive care screenings including cognitive assessments. Health care providers could consider asking patients as young as age 45 years about experiences of worsening memory loss or confusion during visits to initiate discussions about early signs of dementia and strategies to reduce risk and sustain cognitive health.

The findings in this report are subject to at least three limitations. First, sample sizes for some racial and ethnic groups were too small to detect statistical differences, particularly at the state level. Second, BRFSS represents the noninstitutionalized adult population only and therefore cannot be generalized to institutionalized adults. Finally, BRFSS data rely on self-report rather than medical examination records; survey questions about cognitive decline may be subject to recall and social desirability biases and responses may reflect cultural differences. However, the self-perception of cognitive decline has been shown to discriminate preclinical ADRD from normal aging (*7*).

Early detection and diagnosis are important to rule out conditions other than ADRD that might be treatable, and to establish a care plan to manage co-morbid conditions and avoid unnecessary hospitalizations. These potentially avoidable hospitalizations can be both costly and detrimental to quality of life, especially given that some persons with SCD are caregivers for others, which might affect the quality of care provided (*8*). Public health professionals can continue working to improve social determinants of health, conditions in places where people are born, live, learn, work, play, worship, and age that affect risk for developing ADRD (*9*). Although dementia might not be preventable for some, the risk for developing dementia for others can be delayed or reduced through early interventions and public health education including heart-healthy lifestyles, protecting the head from traumatic brain injury, and engaging in social activities (*1*,*2*,*10*). The Building Our Largest Dementia Infrastructure Act (BOLD), charges CDC with strengthening the dementia and dementia caregiving public health infrastructure.*** BOLD Public Health Centers of Excellence on Risk Reduction, Early Detection, and Caregiving are national resources to public health departments in reaching populations at greatest risk for ADRD and their caregivers. States and organizations participating in the National Healthy Brain Initiative and BOLD Public Health Programs^†††^ are strengthening the public health infrastructure utilizing the Healthy Brain Initiative Road Map Series^§§§^ and implementing strategies for reducing dementia in populations with known and widening disparities through programs such as the Chronic Disease Self-Management Program.^¶¶¶^ Public health professionals can use these strategies to reduce risks for dementia in at-risk populations within their jurisdictions.

**Summary**

**What is already known about this topic?**

Subjective cognitive decline (SCD), self-reported memory loss or confusion that is occurring more frequently, might be a symptom of early-stage dementia.

**What is added by this report?**

During 2015*–*2020, approximately 10% of adults aged ≥45 years reported SCD, with the highest prevalence among American Indian or Alaska Native adults (16.7%); prevalence declined with increasing formal educational attainment. Fewer than one half of persons with SCD had discussed their concerns about SCD with a health care professional.

**What are the implications for public health?**

Discussing changes in cognition with a physician is important to identifying potentially treatable conditions, obtaining early diagnosis and detection, developing support systems for caregivers, and establishing a treatment or care plan to help adults remain healthy and independent for as long as possible.

## Footnotes

* Although the term “Alzheimer’s disease” is frequently used, this report uses “Alzheimer disease” in accordance with the American Medical Association Manual of Style, 11th Edition, and *MMWR* style.

†
https://www.cdc.gov/BRFSS/index.html

§ Most recent data collection year for each jurisdiction: 2020 (Alaska, Arizona, Arkansas, California, Delaware, District of Columbia, Hawaii, Idaho, Illinois, Kentucky, Maine, Michigan, Nevada, New Hampshire, New York, North Carolina, Ohio, Oregon, Utah, Vermont, Washington, Wyoming, and Puerto Rico); 2019 (Alabama, Connecticut, Florida, Georgia, Indiana, Iowa, Kansas, Louisiana, Maryland, Minnesota, Mississippi, Missouri, Nebraska, New Mexico, North Dakota, Oklahoma, Pennsylvania, Rhode Island, South Carolina, South Dakota, Tennessee, Texas, Virginia, West Virginia, and Wisconsin); 2018 (New Jersey); 2016 (Massachusetts and Montana); and 2015 (Colorado).

¶ Racial and ethnic categories were classified as American Indian or Alaska Native, non-Hispanic; Asian or Pacific Islander, non-Hispanic; Black or African American, non-Hispanic; White, non-Hispanic; Hispanic or Latino (any race). In addition to these categories, non-Hispanic persons who self-identified as another race or multiracial were classified as other and included in the total.

** https://www.cdc.gov/brfss/annual_data/2020/pdf/2020-response-rates-table-508.pdf;

†† 45 C.F.R. part 46.102(I)(2), 21 C.F.R. part 56; 42 U.S.C. Sect. 241(d); 5 U.S.C. Sect. 552a; 44 U.S.C. Sect. 3501 et seq.

§§
https://www.medicare.gov/coverage/welcome-to-medicare-preventive-visit

¶¶
https://www.cms.gov/Regulations-and-Guidance/Guidance/Manuals/Downloads/bp102c15.pdf#page=268

***
https://www.cdc.gov/aging/bold/index.html

†††
https://www.cdc.gov/aging/awardees/index.html

§§§
https://www.cdc.gov/aging/healthybrain/roadmap.htm

¶¶¶
https://www.cdc.gov/arthritis/interventions/programs/cdsmp.htm
